# Supplementary material for: Using deep learning to detect digitally encoded DNA trigger for Trojan malware in Bio-Cyber attacks
Source: Sci Rep. 2022 Jun 10;12:9631. doi: 10.1038/s41598-022-13700-5 (PMC9186480; doi:10.1038/s41598-022-13700-5)
Supplement: Supplementary file 2 — Supplementary Information 2. [file 41598_2022_13700_MOESM2_ESM.pdf]

Supplementary Material: Appendix A: Generation of ‘steganography +/- ’ DNA

The actual content of the trojan payloads considered for the wetlab experiment is “a.wit:1753b.lab:8492”, which is also used as an example in our previous work [11]. This content is encoded into a DNA sequence with and without considering encryption and steganography. The algorithm used a fragment size of 4, key for the encryption of 60, and steganography key is 3. The encoded DNA sequences are shown in Fig. A.1 and Fig A.2.

```
AGATATAAAGTACGACAGTGCTCTCGGCCCTT
AGATATACAGTACTCAATGGATACATCTCCTT
AGATATAGAGTAATCCATATCGAGAGTGCCTT
AGATATATAGTACGTACGACCGAGATGGCCTT
AGATATCAAGTAATGAATCAATGCATAGCCTT
```

Fig. A.1: Without applying encryption and steganography - normal Trojan payload

```
CTTATGAACGATTGTAATCAAGCAGGAATTCAAGTCTAGGTTTCAATTCTGTCTTCAAATG
TGCAGCTGGTCCTCGGACACAAAATGTTATGTTCAATATGCAGGCGTTACTCTAAAGATGA
TGCATG
CATCTCCCCTAATATTACGGAATTACTCCCAACATGTGAACGGACTGGCAGTCTTCGAGAT
TTTAACCGCATTGCCGCGGCTCGCGCCAAGGTCGCCTCGTATGCCTGGCATATAAAGGTCC
GAAAGG
ACTAGAATCGCCTGTACCGTCACTTCGCCATTAGCCTCCGTCGGGGTGAGAGAATAGTTAA
GCTACCTTCAATCAGCTGCCTCAAAACCGGTAATACACGGTGCCGGATTTGCTCAAATTAA
TTAGCT
CTTGTTATAGATGAGGCCGACCGGTAAAAATAATACTAGGTAACGTGCATTTCTGATGCTT
CATCGCAGAGCCATCTGAGAAAGCGGTGTCCGGATCGCTTCCCGGATCGGCCACCAACCTA
CCGGCG
CGTGGAATCCATGATCCCGTACTCCACTCTGAAGGTGTCGGTCTATCACGGCCGGGGAGCA
AACCGGATTACATGGATCTATTGCAAGTTAAACAGAGGCGGGTGGTCAACCACGACATATT
TGGAGG
```

Fig. A.2: Trojan payload applying encryption and steganography

In these sequences, each line corresponds to a fragment of the trojan payload address (host names and port addresses only). We can insert any encoded line representing a fragment (without breaking) at any position inside an existing DNA sequence (also called our host DNA). However, note that we can not break a encoded line further as it represents a fragment. Furthermore the overlapping (if any) needs to be managed carefully. To summarize, the content of one file can be placed inside one plasmid, where any line can be put at any position (i.e., each line is a part of either the host name or port address of different machines that want to form a connection).

Gene synthesis of DNA fragments with and without applying encryption and steganography

Two separate samples were analyzed; both containing the Trojan payload enabling the attack, but one uses no encryption and steganography (normal Trojan payload) and another uses both encryption and steganography (Trojan payload applying steganography). The sample containing the Trojan payload with steganography, has a length of 640 basepairs (See **Fig. A.2**), while the sample containing the normal Trojan payload has 160 basepairs in length (See **Fig. A.1**). Both DNA sequences were gene synthesised by Eurofins Genomics Europe, Germany, provided in pEX-A128A plasmid vector in lyophilised format and the resulting plasmids named pSTEG and pNOSTEG respectively.

Preparation and transformation of competent cells

E.Coli NovaBlue cells (Novagen, *endA1 hsdR17*($r_{K12}^{-}m_{K12}^{+}$) *supE44 thi-1 recA1 gyrA96 relA1 lac* F'[proA^{+}B^{+} *lacI*^{q} *Z*Δ*M15*::Tn*10* (Tc^{R})]) were inoculated into LB broth and grown overnight at 37°C in a shaking incubator at 250 RPM with adequate aeration. Competent cells were then prepared using the *Mix&Go! E. Coli* Transformation Kit (Zymo Research) as per manufacturer's instructions.

Transformation of competent *E. Coli* NovaBlue cells with pEX-A128 'Trojan payload applying steganography' (pSTEG) and 'Normal Trojan Payload' (pNOSTEG) plasmid DNA was carried out by adding 1 µL of the relevant resuspended plasmid DNA into 50 µL of competent *E. coli* NovaBlue cells, as per *Mix&Go!* kit protocol, and aliquots were spread on pre-warmed LB/Amp (Ampicillin 100 µg/ml) agar plates. A negative control plate was prepared by adding 1 µL of sterile water in place of DNA. Plates were incubated at 37°C overnight. Successfully transformed cells were selected via ampicillin resistance as a selection marker.

Successfully transformed isolated colonies were then inoculated into LB/Amp broth and cultures were incubated until an OD_{600nm} = 2 was reached. OD_{600nm} measurements were taken using the NanoDrop™ 1000 (Thermo Scientific™). Cultures were then concentrated to an OD_{600nm} = 10. Once cultures were at the appropriate OD_{600nm}, plasmid DNA was purified using the Monarch® Plasmid Miniprep Kit (NEB) as per manufacturer's instructions. Plasmid samples were eluted in sterile water and the DNA concentration and quality was assessed using the NanoDrop™ 1000. The presence of the plasmid for each sample was verified using agarose gel electrophoresis (0.8% agarose made with 1xTAE buffer) (**Fig. A.3**).

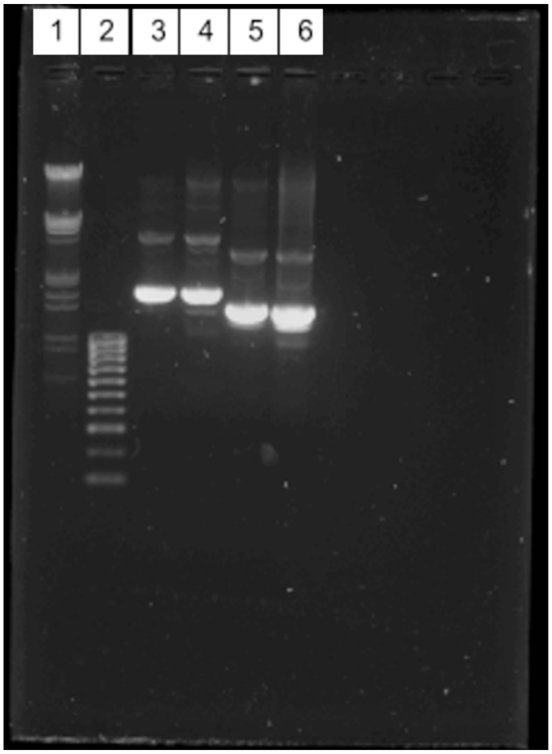

Fig. A.3: Confirmation of plasmid purification using agarose gel electrophoresis. 1: Lambda DNA/EcoR1 plus HindIII ladder. 2: Promega 100bp ladder. 3+4: pSTEG plasmid - Trojan payload applying steganography 5+6: pNOSTEG plasmid - normal Trojan payload.

DNA Sequencing

Samples were sequenced by Eurofins Genomics Europe Sequencing GmbH, Germany. Oligonucleotides used for sequencing were supplied by Eurofins; pEX-For (5'-GGAGCAGACAAGCCCGTCAGG-3') and pEX-Rev (5'-CAGGCTTTACACTTTATGCTTCCGGC-3').

Analysis of sequencing data

Analyses of sequencing data were carried out using a combination of Chromas (v 2.6.6) and MEGA-X (v 10.2.6). Sequencing chromatogram quality was first assessed using Chromas. Sequence alignments were performed using the CLUSTALW algorithm in MEGA-X. Following successful alignment of DNA sample sequence with reference sequence, the sequences were trimmed in Chromas to highlight the ‘Trojan payload applying steganography’ DNA and ‘Normal Trojan payload’ DNA only for analysis. (Sample sequencing results are shown in Fig A.4).

Sequencing Results

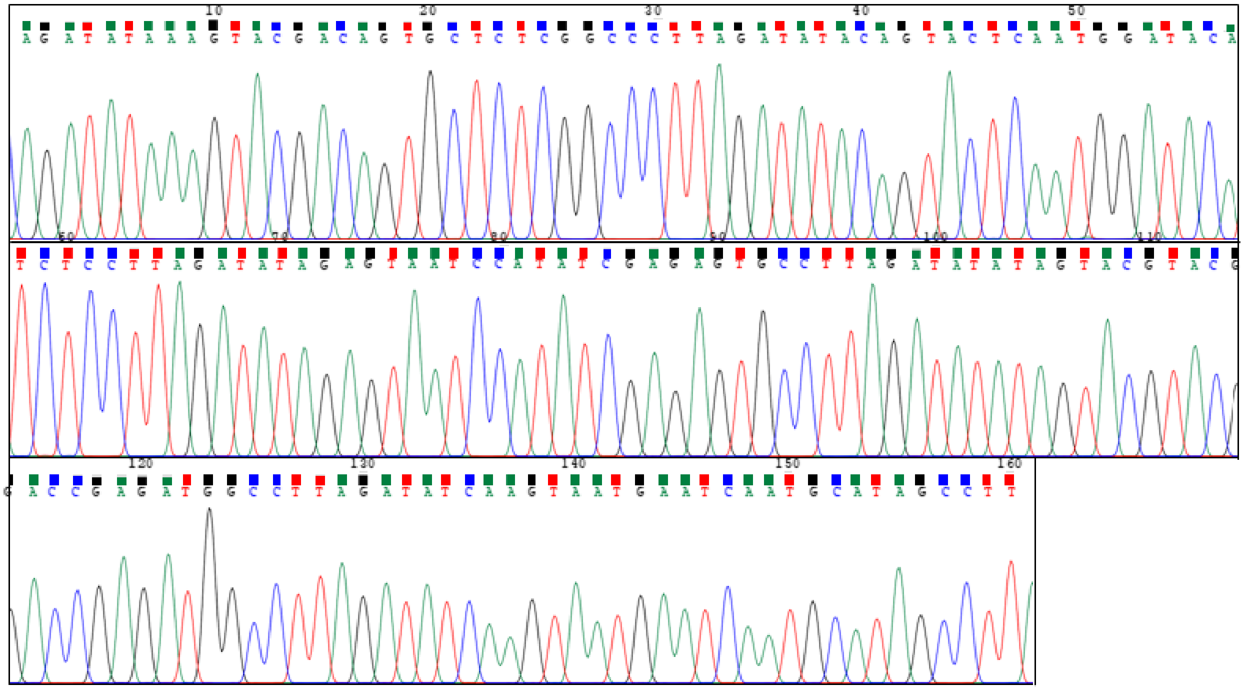

Fig. A.4: Sample sequencing chromatogram from pNOSTEG with 60bp DNA sequence region for Trojan payload address without encryption and steganography applied visible.

Author Contribution

Mr. Mohd Siblee Islam is the primary author of the article. Mr. Islam was responsible for developing the software code used to perform computational experiment, executing the experiments, analysing and interpreting the results presented in this article, writing the manuscript.

Dr. Stepan Ivanov was responsible for overseeing and directing computational experiments presented in this article. Specifically, Dr. Ivanov contributed to the development of the proposed steganography technique, where he proposed the dynamic programming technique for finding an optimal location for the payload for malicious activity to be injected into the host DNA. Dr. Ivanov assisted Mr. Islam in writing the manuscript.

Dr. Sasitharan Balasubramaniam was the main scientific driver behind the experiments presented in the article. Due to his multidisciplinary background, Dr. Balasubramaniam identified the possibility for E.Coli bacteria to be used as carriers of malicious DNA on-purposed engineered as part of a Trojan attack. That was the starting point for the research presented in the article. Subsequently, Dr. Balasubramaniam directed and oversaw the experiments conducted in this research.

Dr. Lee Coffey planned and executed the wet lab experiments, including gene synthesis design, cloning and recombinant plasmid DNA purification.

Dr. Srivatsan Kidambi was responsible for providing expertise in methods for handling DNA based samples and background for DNA packaging/carrying.

Ms. Jennifer Drohan prepared the DNA samples for sequencing and carried out sequence analysis of the DNA fragments in order to verify sequence identity and fidelity.

Dr. Witty Sri-saan was the scientific driver behind the DNN analysis for the DNA strands with the injected code, as well as the development of the hacking scenarios.

Dr. Hamdan Awan was responsible for the analysis of the data in the results section and in particular the analysis on performance based on variations in parameters.

Data Availability Statement

All data used in the manuscript are available in the Addgene repository (https://www.addgene.org/), where the DNA sequences of type plasmid of E.Coli bacteria are collected for our experiments using web scraping. This data is also available as a supplementary document (all_plasmid_dna.txt). The Programming code developed to conduct the experiments (also the scripts for the data collection from Addgene) is freely available in the publicly available git repository at the following URL: https://github.com/sibleeislam/trojan-malware-in-bio-cyber-attacks. For any further query related to data availability please contact using the email of the primary author (*sibleeislam@gmail.com*) of the manuscript.

Competing Interests Statement

None of the authors or their respective organizations/research groups have any financial or otherwise interests that could affect or compromise findings of the research presented in this manuscript. The research presented in this article was carried out in strict accordance to the rules of research ethics and conduct.

Artwork Statement

Artwork on Fig. 2 and 4 of the article was created by Mohd Siblee Islam using free Draw.io software and free icons available on the web. Artwork on Fig. 1 and 3 of the article was created by Dr. Ivanov and Mohd Siblee Islam using free Draw.io software and free icons available on the web.
